# Learning from animals: How to Navigate Complex Terrains

**DOI:** 10.1371/journal.pcbi.1007452

**Published:** 2020-01-09

**Authors:** Henghui Zhu, Hao Liu, Armin Ataei, Yonatan Munk, Thomas Daniel, Ioannis Ch. Paschalidis

**Affiliations:** 1 Center for Information and Systems Engineering, Boston University, Boston, Massachusetts, United States of America; 2 College of Control Science and Engineering, Zhejiang University, Hangzhou, China; 3 Department of Biology, University of Washington, Seattle, Washington, United States of America; 4 Department of Electrical and Computer Engineering, Division of Systems Engineering, and Department of Biomedical Engineering, Boston University, Boston, Massachusetts, United States of America; University of Cincinnati, UNITED STATES

## Abstract

We develop a method to learn a bio-inspired motion control policy using data collected from hawkmoths navigating in a virtual forest. A Markov Decision Process (MDP) framework is introduced to model the dynamics of moths and sparse logistic regression is used to learn control policy parameters from the data. The results show that moths do not favor detailed obstacle location information in navigation, but rely heavily on optical flow. Using the policy learned from the moth data as a starting point, we propose an actor-critic learning algorithm to refine policy parameters and obtain a policy that can be used by an autonomous aerial vehicle operating in a cluttered environment. Compared with the moths’ policy, the policy we obtain integrates both obstacle location and optical flow. We compare the performance of these two policies in terms of their ability to navigate in artificial forest areas. While the optimized policy can adjust its parameters to outperform the moth’s policy in each different terrain, the moth’s policy exhibits a high level of robustness across terrains.

## Author summary

Many animals exhibit a remarkable ability to navigate in complex forest terrains. Can we learn their navigation strategy from observed flying trajectories? Further, can we refine these strategies to design UAV/drone navigation policies in dense cluttered terrains? To that end, we propose a method to analyze data from hawkmoth flight trajectories in a closed-loop virtual forest and extract the navigation control policy. We find that moths rely heavily on optical flow rather than detailed information on the location of obstacles around them. We also develop a method to refine the hawkmoth control policy to be used by autonomous aerial vehicles in a cluttered environment. We find that integrating both obstacle location information and optical flow improves navigation performance.

## Introduction

Moths and other animals are experts in navigating complex forest terrains [1, 2]. Recent work in [3] experimented with hawkmoths (*Manduca sexta*) playing “video games” of navigation. These experiments revealed that when navigating through a virtual forest, moths determine their route ahead of time depending on how much of the forest they can see. Compared with a distribution of trajectories that are randomized via resampling, [3] suggests that moths respond to the external stimuli and follow a deliberate, goal-directed navigation path.

Such behaviors could inspire novel data-driven algorithms for control of autonomous vehicles performing complex tasks, including collision avoidance [4], navigation [5] and *Simultaneous Localization And Mapping (SLAM)* [6]. Problems of this type are typically formulated as dynamic optimization problems which can, in principle, be solved by dynamic programming techniques [7]. Unfortunately, however, such methods do not lead to practical policies for problems of realistic size due to the well known curse of dimensionality (too many “states” a vehicle can be at and too many feasible control actions at each state).

An alternative approach is learning from demonstration, which involves modeling all the individual actions, integrating all possible features leading to specific actions, and learning the right sequence of actions either by observing an expert performing the whole task or through reinforcement learning [8]. Bio-inspired control and decision rules have received significant attention in the literature. Some efforts focus on low-level control components, including optical flow [4, 9] and echolocation [10], whereas others seek to mimic brain dynamics by neural network models [11] or develop related reinforcement learning models [12]. While these approaches capture low-level control for specific tasks and/or the (neural) architecture that enables such control, they may not generalize to different or more complex tasks due to the lack of a higher-level planning strategy that adapts to these tasks.

In contrast, our primary objective is not necessarily to learn a control policy used by animals (moths) to fly in a given terrain. Rather, we want to capture a rich enough parametric policy structure and learn specific parameters corresponding to the observations at our disposal. We also want to develop a method that would allow us to adapt these parameters to fit an autonomous vehicle operating in a different terrain, thereby obtaining a *bio-inspired* policy instead of a policy that merely mimics the observed animal.

The remainder of the paper is organized as follows. The Materials and Methods section presents the experimental setup that was used to obtain moth flight data, introduces a parametric policy structure in an MDP setting, discusses how to estimate policy parameters from data, and outlines an actor-critic method to refine the policy by optimizing a long-term average performance over policy parameters. The Results section presents our results by analyzing the data; specifically, we compare the estimated moth policy and the refined policy in different environments. We discuss the results in the Discussion section.

## Materials and methods

### Experimental setup

**Fig 1:**
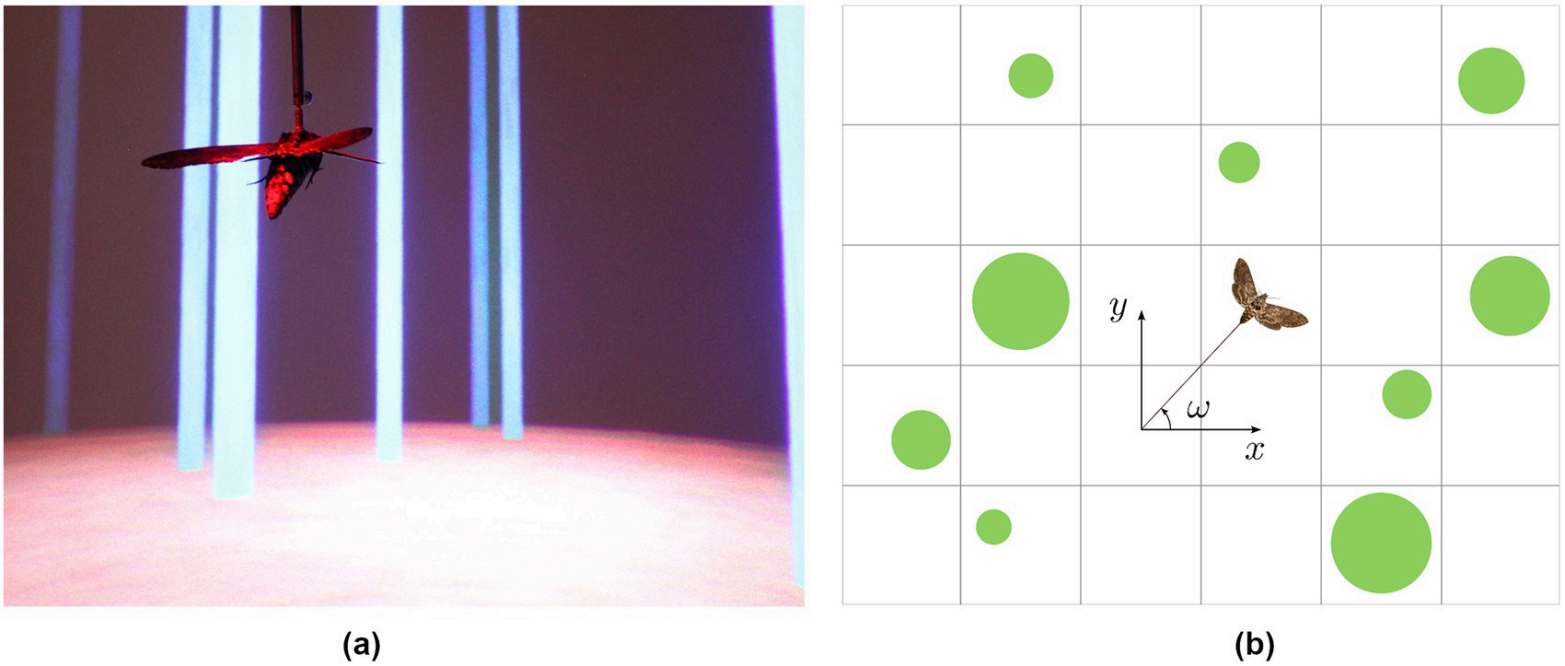
An image of the setup for the moth’s navigation experiment and a grid discretizing the moth’s position. (a) The experimental setup. The moth is attached to a rod and views light bars corresponding to forest trees. (b) Top view of a hawkmoth in a virtual (discretized) forest.

The experimental setup is based on earlier work in [3] which constructed a virtual forest to study the navigational behavior of the hawkmoth *Manduca sexta* (see Fig 1). There are 100 virtual trees in the forest with average diameter 0.0811 (m). The trees spread out, covering a rectangular area with length 139.98 (m) and width 139.69 (m). A total of 8 moths were used in the experiments. The moths were raised in a lab colony, and they were not fed as adults. They were used two days after eclosion (emergence from the pupae). Each moth used in the experiment was connected to a torque meter through a connecting rod and was placed in front of a large screen which displayed the projection of a forest. This virtual forest was then moved at a constant speed (2 m/s) relative to the moth and reaction of the moth was measured through the torque meter in the yaw direction. These measurements reflected the moth’s attempt to change its direction and they were applied by changing the viewing angle of the virtual forest. The position, heading, and the control effort applied to the torque meter were recorded at a rate of 60 Hz. The visual field of the moth was also hindered by introducing virtual fog to the forest; this was done by reducing the contrast of the trees against the background, thereby limiting the visibility range of the animals. Each of the moths performed 5 trials under 5 different levels of fog density: 0.83, 3.33, 6.67, 13.33, and 26.67.

### The unicycle model

To model the behavior of moths, we start by discretizing the experimental forest area into a discrete set of points. Since the moth is physically connected to the torque meter, its movement is constrained and can only perform a level flight. Therefore, we can discretize the space by generating a grid over the level-flight plane. We also discretize the headings that moths can assume. Let (*x*, *y*, *ω*) describe a three-tuple discretized space corresponding to the position (*x*, *y*) and heading *ω* of the moth in the forest (see Fig 1(b)). A simple model describing the movement of the moth can be derived from the unicycle model below,

$$\dot{x}=v\;\text{cos}\;\omega ,\;\dot{y}=v\;\text{sin}\;\omega ,\;\dot{\omega }=\kappa ,$$ (1)

where *v* and *κ* denote the linear speed of the moth and its angular velocity, respectively. The above system essentially describes the kinematics of a particle with zero mass. This model provides an approximation to the flight of the moth due to its relatively small size and weight.

A given flight trajectory can also be discretized in time and space. First, the trajectory is discretized in time, which reduces a continuous trajectory into a path going through a series of discrete points. Then, each discrete point of the flight trajectory is mapped onto a discrete point on the discretized space (see Fig 1(b)). We will discuss later in the Results Section the discretization we selected. Clearly, the distance between the flight-trajectory discrete points and their corresponding points on the grid gets reduced as the grid becomes finer. However, a finer grid results in much higher computational cost and may prove to be intractable. A coarser grid, on the other hand, results in discrepancies between the true point on the discretized trajectory and its corresponding point on the grid. This leads to ambiguities when calculating the next position of the moth from the unicycle model, suggesting a probabilistic transition model. A probabilistic model can also account for other types of uncertainties, including air resistance, differences in visual perception and behavior between multiple test animals, and other factors that may affect the moth’s next position. Since, however, the next position only depends on the current position and the control input *κ* (assuming *v* is constant), we can model the flight of the moth using a *Markov Decision Process (MDP)*. In the following subsection, we formulate the moth’s navigation problem in an MDP setting and introduce the parameterized policy structure we will use.

### Moth policy structure

We consider a discrete-time MDP with a finite state-space X and an action space U [7], which are discretized from the continuous-time unicycle model. Let $x_{k}\in X$ and $u_{k}\in U$ be the state of the system and the action taken at time *k*, and let **x**_0_ be the initial state. Specifically, the state **x** consists of the discretized moth coordinates (*x*, *y*) and heading *ω*, and the action *u* is the discretized angular velocity *κ*. Selecting a discretized angular velocity *u* at time *k*, and given its current position and heading **x**_*k*_ = (*x*_*k*_, *y*_*k*_, *ω*_*k*_), the moth can essentially determine its position and heading at time *k* + 1, **x**_*k*+1_ = (*x*_*k*+1_, *y*_*k*+1_, *ω*_*k*_), which can be obtained from the unicycle model (1) assuming a constant speed *v* and mapping the outcome to a discrete state. Notice that the cardinality of the state space can be very large. For example, using a 608 × 609 grid environment with 72 possible heading directions results in an MDP with a state space of 26, 659, 584 states!

Let *p*(**x**_*k*+1_|**x**_*k*_, *u*_*k*_) denote the probability that the next state is **x**_*k*+1_, given the current state **x**_*k*_ and the action taken *u*_*k*_. Let *g*(**x**_*k*_, *u*_*k*_) be the one-step reward at time *k* when action *u*_*k*_ is applied at state **x**_*k*_. A *Randomized Stationary Policy (RSP)* is a mapping *μ* that assigns to each state x∈X a probability distribution for taking an action u∈U. We consider the following parameterized policy:

$$\mu \left(u|x;\theta \right)=\frac{\text{exp}(\theta^{\prime }\phi \left(x,u\right))}{\sum_{v\in U}\text{exp}\left(\theta^{{}^{\prime }}\phi \left(x,v\right)\right)},$$ (2)

where $\phi \left(x,u\right)\in R^{n}$ is a vector of state-action “features” and ***θ*** = (*θ*_1_, …, *θ*_*n*_) is an *n*-dimensional parameter assigning weights to the features. (We use prime to denote transpose, so ***θ***′*ϕ*(**x**, *u*) is the dot product of two column vectors. In general, all vectors are column vectors and denoted by boldfaced lowercase letters.) Notice that this policy selects actions by only considering the feature vector *ϕ*(**x**, *u*) and not the state-action pairs (**x**, *u*) directly. More specifically, each element of *ϕ*(**x**, *u*) = (*ϕ*_1_(**x**, *u*), …, *ϕ*_*n*_(**x**, *u*)) corresponds to a feature of the state-action space used in selecting action *u* at state **x**. One can interpret −***θ***′*ϕ*(**x**, *u*) as an “energy” function and view Eq (2) as representing a Boltzmann distribution for selecting action *u*.

The MDP model assumes an one-step reward function *g*(**x**_*k*_, *u*_*k*_) associated with state-action pair (**x**_*k*_, *u*_*k*_). We will discuss later in the Results Section how such a function can be defined based on the moth trajectory data. The objective of the MDP is to maximize an expected average reward defined by

$$R=\underset{K\to \infty }{\text{lim}}E[\frac{1}{K}\sum_{k=0}^{K-1}g\left(x_{k},u_{k}\right)],$$ (3)

where the expectation E[·] is taken with respect to the stationary distribution of the Markov chain {(**x**_*k*_, *u*_*k*_)}. We next present the various state-action features in *ϕ*(**x**, *u*) that we will use to capture the moth’s policy. We note that not all these features are equally important in determining how the moths move; their relative weights will be determined based on the trajectory data.

#### Obstacle spatial density

**Fig 2:**
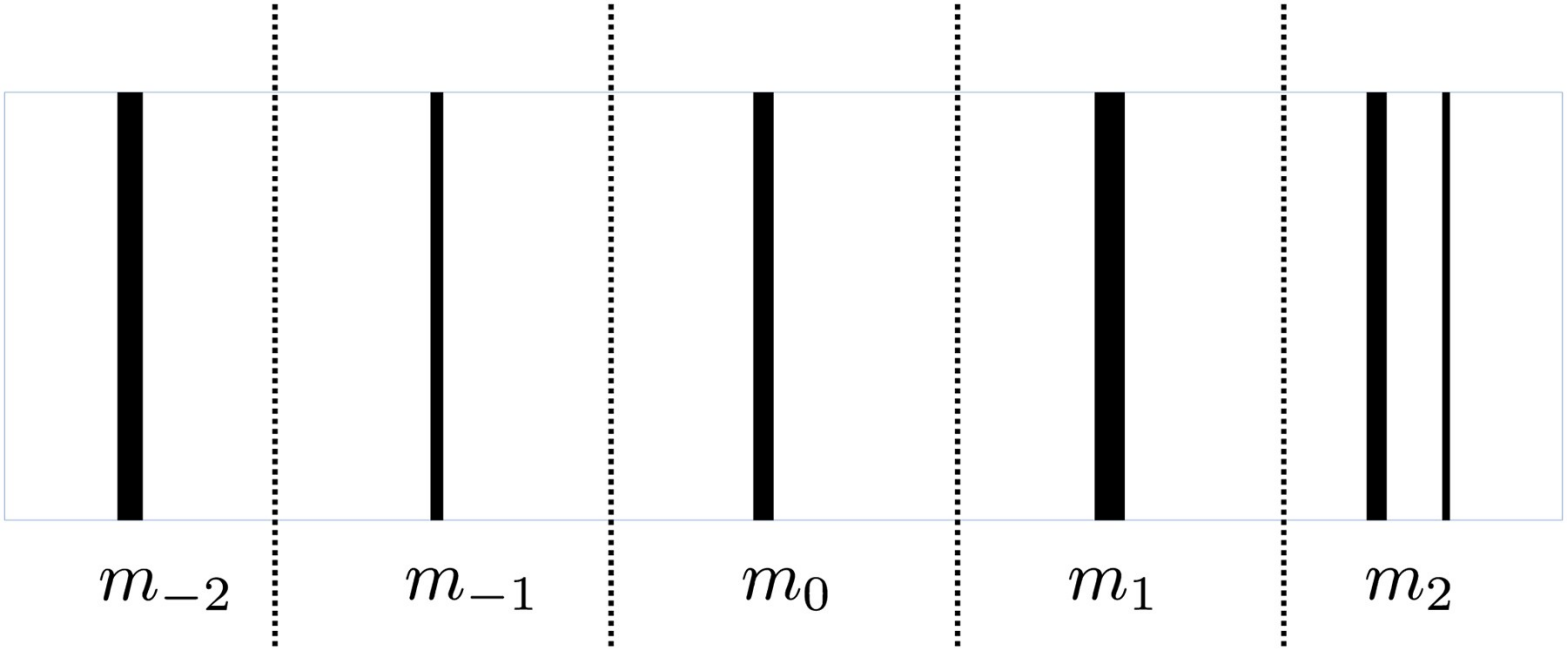
Visual field segmentation for *m* = 2.

The field of view of the moth is divided into 2*m* + 1 equal segments, with each segment corresponding to the desired next heading of the moth, which is determined by the control action *u*; specifically, we have |U|=2m+1. The entire field of view has a range of *π*/2. The obstacle spatial density is calculated by projecting the forest trees onto the field of view associated with the current state of the moth. Due to the fog in the visual forest, the distance of perception is limited by the fog level. Also, since the eye has a finite resolution, any projection with a value smaller than a preset angular resolution is discarded (see Fig 2). We define an obstacle spatial density-based feature *V*(**x**, *u*) equal to the percentage of area of the segment corresponding to desired heading *u* that is covered by trees. We also introduce “filtered” versions of this feature. Specifically, we define a threshold *p* ∈ (0, 1]. We set a feature value *V*_*p*_(**x**, *u*) = 1 if more than a fraction *p* of the segment corresponding to heading *u* is covered by trees; otherwise *V*_*p*_(**x**, *u*) = 0. We can include multiple such features, each with a different value of *p*. In our results we use two thresholds: *p* = 0 and *p* = 0.5.

#### Optical flow

In a bio-inspired context, optical flow is defined as the change of light in the visual imagery projected onto the retina caused by relative movements between the eyeball and the scene [13]. It is widely believed that optical flow plays an important role in the vision systems of animals, including the hawkmoths [14]. Accurate estimation of optical flow, which is represented as a vector field, is a computationally demanding task. Because in our experimental setting the movements of moths are constrained to be in a horizontal plane, we ignore the light difference in vertical direction, i.e., the visual inputs of hawkmoths can be represented as an one-dimensional function of position.

Reference [15] introduces a simple way to estimate optical flows for the one-dimensional case. Let *V*_*k*_(*u*) and *V*_*k*+1_(*u*) be one-dimensional signals at two consecutive discrete time epochs *k* and *k* + 1. By the definition of optical flow, *V*_*k*+1_(*u*) should be generated by translating *V*_*k*_(*u*), i.e., *V*_*k*+1_(*u*) = *V*_*k*_(*u* − *s*) where *s* denotes the translation. Expanding *V*_*k*_(*u* − *s*) using Taylor’s series, we obtain

$$V_{k+1}\left(u\right)=V_{k}\left(u-s\right)=V_{k}\left(u\right)-s\frac{dV_{k}\left(u\right)}{du}+O(s^{2}\frac{d^{2}V_{k}\left(u\right)}{du^{2}}).$$

As a result, the difference between the two signals is

$$V_{k}\left(u\right)-V_{k+1}\left(u\right)=s\frac{dV_{k}\left(u\right)}{du}-O(s^{2}\frac{d^{2}V_{k}\left(u\right)}{du^{2}}).$$

Ignoring higher order terms, we can approximate the optical flow as

$$\hat{s}\left(u\right)=\frac{V_{k}\left(u\right)-V_{k+1}\left(u\right)}{(dV_{k}\left(u\right))/du}.$$ (4)

For a higher dimensional vector field **f**, a common practice is to discretize the signal into a discrete vector field. Let **f**_*k*_ = (*V*_*k*_(1), …, *V*_*k*_(2*m* + 1)), and **f**_*k*+1_ = (*V*_*k*+1_(1), …, *V*_*k*+1_(2*m* + 1)) be the discretized obstacle spatial density feature perceived by the moth at time *k* and *k* + 1, where 2*m* + 1 is the number of discretization levels and 1, …, 2*m* + 1 index the visual segments from left to right (see Fig 2); we suppress the dependence of *V*_*k*_(*u*) on the position **x**_*k*_ for ease of notation. If we approximate the derivative in (4) with the corresponding first-order difference, the optical flow can be represented as **s** = (*s*(1), …, *s*(2*m* + 1)), where

$$s\left(u\right)=\frac{V_{k}\left(u\right)-V_{k+1}\left(u\right)}{V_{k}\left(u+1\right)-V_{k}\left(u\right)},\;u=1,\ldots ,2m.$$

Since the derivative for the last element is missing, we set *s*(2*m* + 1) = *s*(2*m*). The optical flow feature is defined equal to *s*(*u*) at position **x** and heading *u*, where, as mentioned earlier, the dependence of *s*(*u*) on **x** is suppressed.

#### Control history

This feature reflects the willingness of the moth to fly on a smooth path instead of actively searching or avoiding trees. This is modeled by taking an average over the last *q* control actions. For the case where *q* = 1, this feature simply corresponds to the last control action. Note that adding past control actions to features will require the MDP to be modified by augmenting the state space to include all combinations of the original states and the last *q* control actions.

#### Energy

We assume the amount of energy for continuous straight flight is different from the energy required for turning. For example, a sample behavior may show that the moth prefers to maintain a straight flight and make sharp turns when needed, or it may show that it attempts to avoid making sharp turns. These behaviors are modeled using different energy levels required for the commanded turn signal. In this paper, the energy feature *ϕ*(**x**, *u*) is defined as the amount of the torque needed to select the next heading *u* at state **x**. Specifically, suppose that the moth is flying straight; then, there is no torque needed and the energy spent at this time is zero. On the other hand, if the moth wishes to change its heading, then it needs to expend some energy proportional to the magnitude of the turn.

### Learning the moth control policy

The parameters ***θ*** of the moth control policy are estimated using *logistic regression*. We introduce appropriate regularization in logistic regression to induce sparsity, thus identifying the most essential features driving the moth’s movements consistent with the data. We also discuss the optimization methods we use in order to solve the sparse logistic regression problem. There is theoretical evidence that estimation of the control parameters using sparse logistic regression is robust to noise in the data [16] and leads to favorable regret of the estimated policy [17, 18].

To formulate the estimation problem, let $D=\{\left(x_{1},u_{1}\right),\ldots ,\left(x_{N},u_{N}\right)\}$ represent the experimental observations, where **x**_*k*_ is the state and *u*_*k*_ the control action applied by the moth at state **x**_*k*_. We assume that these observations are independent and identically distributed (i.i.d.). As we will see, we construct D from multiple moth trajectories, assuming that in all trajectories (potentially involving different animals) the control policy used by the moths is the same. The negative log-likelihood (NLL) of the experimental observations in D is

$$\begin{array}{cc} \text{NLL}(\theta ) & =\sum_{(x_{i},u_{i})\in D}\left[-\text{ln}\;\mu \left(u_{i}|x_{i};\theta \right)\right]=\sum_{i=1}^{N}\left[-\text{ln}\;\mu \left(u_{i}|x_{i};\theta \right)\right]\\ & =\sum_{i=1}^{N}\text{ln}(\sum_{u\in U}\text{exp}(\theta^{\prime }\phi (x_{i},u))-\sum_{i=1}^{N}\theta^{\prime }\phi \left(x_{i},u_{i}\right). \end{array}$$ (5)

We can estimate a control policy parameter vector θ* in Eq (2) consistent with the data D by minimizing the negative log-likelihood (5), namely,

$$\theta^{*}=\text{arg}\;\underset{\theta }{\text{min}}\;\text{NLL}\left(\theta \right).$$ (6)

In many situations, we have numerous candidate features, and need to identify a small set of important features. Similar to the LASSO method [19], we can add an *ℓ*_1_-norm regularization term to induce sparsity and determine which features are of the most importance. This results in the following *sparse logistic regression* objective

$$\begin{array}{c} \underset{\theta }{\text{min}}\;{\text{NLL}}_{\text{sp}}\left(\theta \right)=\underset{\theta }{\text{min}}\{\text{NLL}\left(\theta \right)+{\lambda ∥\theta ∥}_{1}\}, \end{array}$$ (7)

where ∥⋅∥_1_ is the *ℓ*_1_-norm of the parameter vector ***θ*** and λ some scalar penalty parameter.

The objective function in (7) is convex, and its subgradient can be obtained in closed form according to Eq. (S.3) in S1 Text. The optimization problem (7) can be solved using many numerical optimization methods, such as the quasi-Newton BFGS method [20]. The details can be found in S1 Text.

### Refining the moth control policy

The learned parametric policy can serve as a good starting point to find a policy adapted to navigation of a UAV in a potentially different terrain. We assume we have an one-step reward function *g*(**x**_*k*_, *u*_*k*_) driving navigation decisions. The objective is to maximize the average reward (cf. (3)) over the control policy parameter vector ***θ***.

When the number of states in the MDP is large, standard methods become intractable due to the well-known curse of dimensionality. One approach to solve this problem is to use an actor–critic algorithm [21]. This paper uses a modified version of a *Least-Squares Temporal Difference (LSTD)* actor-critic algorithm developed in [22].

Denote by ***ψ*** the gradient of the log-likelihood of control *u* at state **x**,

$$\psi_{\theta }\left(x,u\right)=\nabla_{\theta }\;\text{ln}\;\mu \left(u|x;\theta \right).$$ (8)

The LSTD algorithm is given in Algorithm 1, where we use the following stepsizes *ζ*_*k*_, Γ(**r**) and *η*_*k*_:

$$\begin{array}{c} \gamma_{k}=\frac{1}{k},\;\Gamma \left(r\right)=\{\begin{array}{cc} \frac{D}{∥r∥},\; & \text{if}\;∥r∥>D,\\ 1,\; & \text{otherwise}, \end{array}\;\text{and}\;\eta_{k}=\frac{c}{k\text{ln}k}, \end{array}$$

with *D* and *c* being some positive constants.

**Algorithm 1**: LSTD actor-critic algorithm. In this algorithm, *ζ*_*k*_ controls the critic step-size, Γ(**r**) and *η*_*k*_ together control the actor step-size, *ρ* ∈ (0, 1) is a discounting factor taken close to 1, and *ϵ* is a parameter controlling how close to a stationary point we wish to converge.

**Initialization**: Initialize **z**_0_, **A**_0_, **b**_0_, and **r**_0_ with zero entries. Let ***θ***_0_ take the value obtained from the sparse logistic regression and set the initial estimate of the average reward to *a*_0_ = 0.0972, the expected average reward of the MDP under that policy. Choose the initial state **x**_0_ randomly. Let *k* = 0.

**while** ∥*θ*_*k*_ − *θ*_*k*−1_∥ > *ϵ* **do**

 **State update**:

 Use the RSP ***θ***_*k*_ to generate the control *u*_*k*_. Find the next state **x**_*k*+1_.

 **Critic update**:

$$\begin{array}{cc} a_{k+1} & =a_{k}+\zeta_{k}\left[g\left(x_{k},u_{k}\right)-a_{k}\right],\\ z_{k+1} & =\rho z_{k}+\phi \left(x_{k},u_{k}\right),\\ b_{k+1} & =b_{k}+\zeta_{k}\left[\left(g\left(x_{k},u_{k}\right)-a_{k}\right)z_{k}-b_{k}\right],\\ A_{k+1} & =A_{k}+\zeta_{k}[z_{k}{\left(\phi \left(x_{k+1},u_{k+1}\right)-\phi \left(x_{k},u_{k}\right)\right)}^{\prime }-A_{k}],\\ r_{k+1} & =A_{k}^{-1}b_{k}. \end{array}$$

 **Actor update**:

$$\theta_{k+1}=\theta_{k}+\eta_{k}\Gamma \left(r_{k}\right){\left(\phi (x_{k},u_{k})\right)}^{\prime }r_{k}\psi_{\theta_{k}}\left(x_{k+1},u_{k+1}\right).$$

 **Time counter update**: *k* = *k* + 1.

**end**

The LSTD actor-critic algorithm is a stochastic gradient method for maximizing the average reward. Hence, it can not be guaranteed to obtain a global optimal solution. Convergence results [22, 23] establish that it converges to a neighborhood of a stationary point of the expected average reward with probability one (w.p.1).

## Results

### The moth control policy

**Fig 3:**
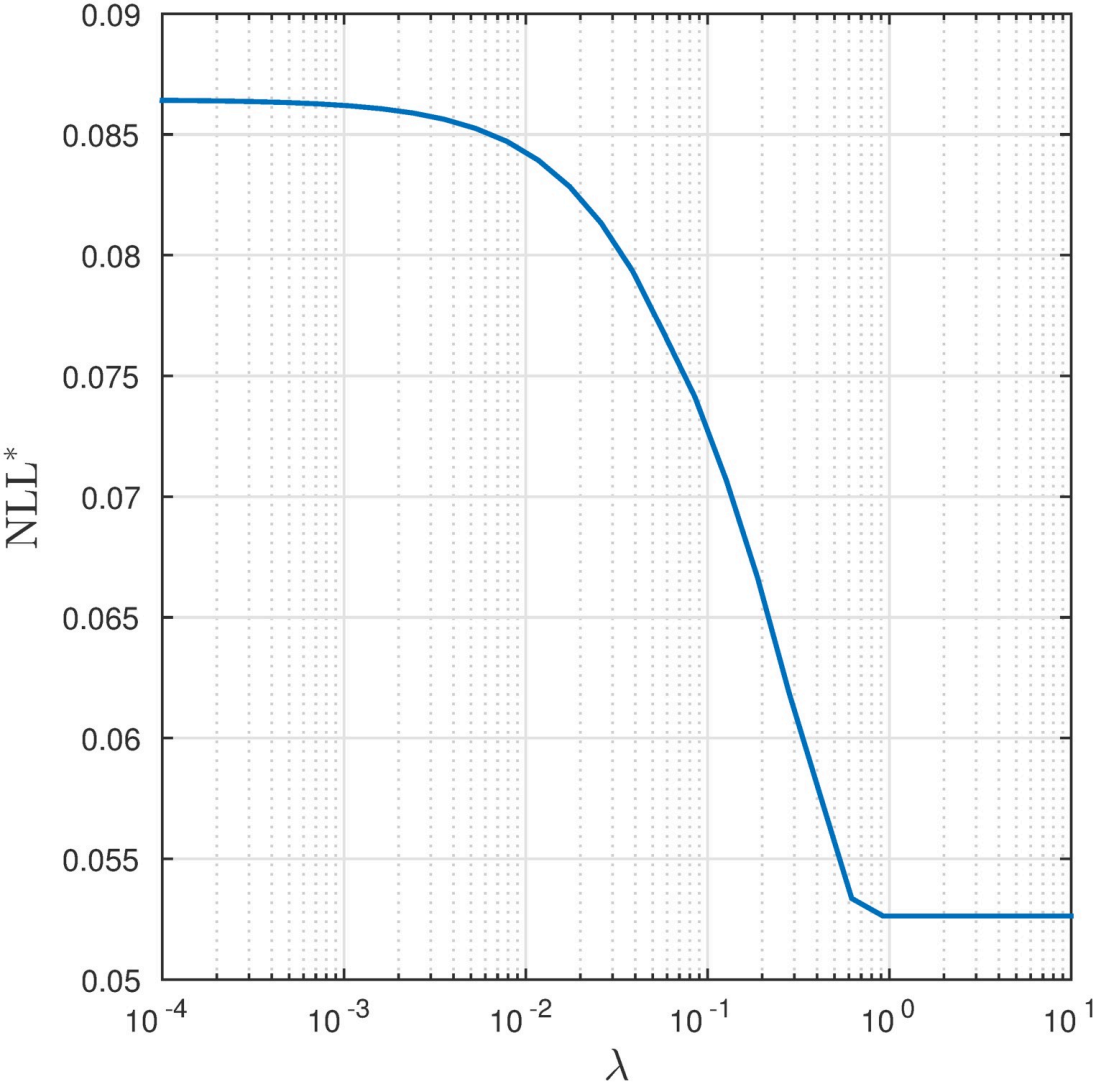
Results of sparse logistic regression. The figure plots the negative log-likelihood function under different regularization penalties λ.

**Fig 4:**
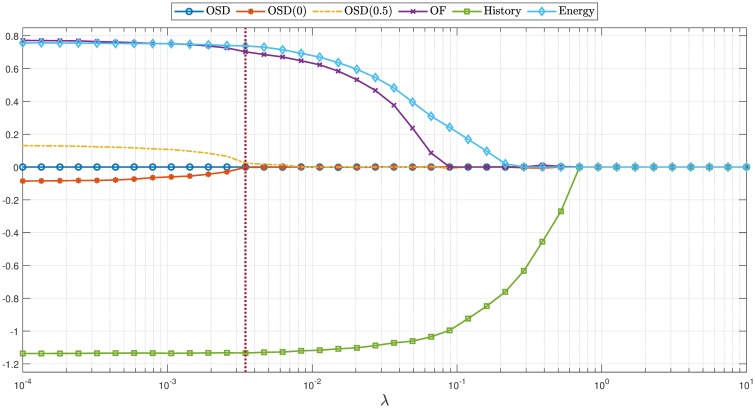
Results of sparse logistic regression. The plot shows regressed parameters *θ* under different λ from empirical flight trajectories. OSD is the obstacle spatial density feature; OSD(0) and OSD(0.5) correspond to OSD features with thresholds 0 and 0.5, respectively. OF is the optical flow feature. Cross-validation suggests λ = 0.0053 (identified by a dotted vertical line in the plot) is the best regularization parameter.

In total, 62,651 data points were collected for regression, corresponding to multiple animals and trajectories. (The data and the code producing the results in this paper are available at https://github.com/noc-lab/moth_navigation). We use 80% of the data in D as training data to regress (7) with different λ’s, and use the remaining 20% of the data to cross-validate the regressed model. The training and validation results are shown in Figs 3 and 4. Since the *ℓ*_1_ regularization selects features according to their importance, Fig 4 indicates optical flow, control history and energy to be the most important features, whereas features related to obstacle spatial density are of the least importance.

After selecting λ, we use all the data to obtain an optimal ***θ***. The results are shown in Table 1. Since all the features are normalized, the magnitude of each component of ***θ*** reflects the importance of the corresponding feature.

**Table 1:** The parameters *θ* of the moth policy and the refined policy. OSD is the obstacle spatial density feature; OSD(0) and OSD(0.5) denote OSD features with thresholds 0 and 0.5, respectively. OF is the optical flow feature. Percentages are the absolute value of weights normalized by the *ℓ*_1_ norm of the weight vector. Reward is the expected average reward (3) for the regressed policy and the optimized policy.

	The moth policy		The refined policy	
	Weights	Percentages	Weights	Percentages
OSD	0.0000	0.0000	1.2590	0.0090
OSD(0)	-0.0817	0.0286	38.8491	0.2770
OSD(0.5)	0.1234	0.0432	-8.6711	0.0618
OF	0.7644	0.2674	66.5153	0.4742
History	-1.1349	0.3970	-9.2061	0.0656
Energy	0.7546	0.2639	15.7742	0.1125
Reward	0.1006		0.1668	

### One-step reward function

To refine the moth control policy and adapt it for a UAV operating in a similar forest terrain, we need to define an one-step reward function. The purpose is to capture important objectives of UAV navigation so that maximizing average reward induces an appropriate UAV navigation policy. We assume that the one-step reward function only depends on the position of the vehicle/moth. For a grid point centered at coordinate (*x*, *y*), the un-normalized one-step reward is defined as

$$\hat{g}\left(x,y\right)=\sum_{i=1}^{M}f_{\alpha ,\beta }(\frac{d_{i}\left(x,y\right)}{r_{i}}),$$ (9)

where *d*_*i*_(*x*, *y*) is the distance between (*x*, *y*) and the *i*th tree, *r*_*i*_ is the diameter of the *i*th tree, and *M* is the number of trees in the forest. Notice that the reward is only a function of the location (*x*, *y*) and not the heading *ω*. The function *f*_*α*,*β*_(*s*) is a polynomial function of 0 ≤ *s* ≤ *γ* with parameters *α* and *β* satisfying the following properties:

$$f_{\alpha ,\beta }\left(0\right)=-0.1,\;f_{\alpha ,\beta }\left(\alpha \right)=0,\;f_{\alpha ,\beta }\left(\frac{\alpha +\gamma }{2}\right)=P,\;f_{\alpha ,\beta }\left(\gamma \right)=0.$$ (10)

**Fig 5:**
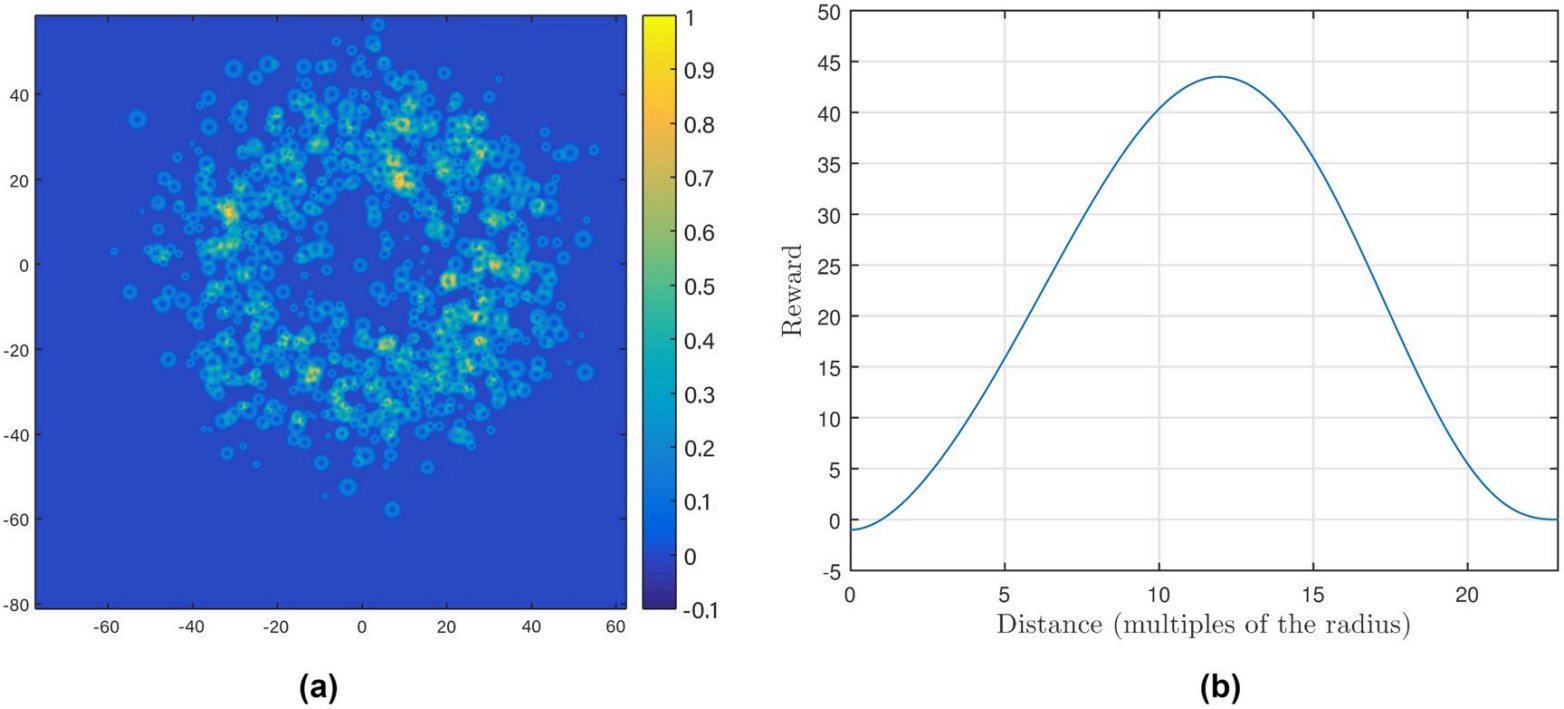
(a) The one-step reward plotted in the experimental forest; the plot is centered and the units of the horizontal and vertical axes are meters, while the color indicates the relative value of the reward. (b) The function *f*_*α*,*β*_ defined in Eq (10) with *α* = 1 and *β* = 1; the horizontal axis indicates distance in multiples of the tree radius and the vertical axis the value of the function.

We use *γ* = *β*/sin(*π*/72). Fig 5(b) shows an example of *f* with *α* = 1, *β* = 1 and P=43.5. Here, *f*_*α*,*β*_(*s*) ≤ 0 if 0 ≤ *s* ≤ *α* and *f*_*α*,*β*_(*s*) ≥ 0 when *α* ≤ *s* ≤ *γ*. Moreover, we set *f*_*α*,*β*_(*s*) = 0 when *s* ≥ *γ*. We then normalize the reward function $\hat{g}$ to be in [−0.1, 1]. The one-step reward plotted in the experimental forest used to generate the moth data is shown in Fig 5(a).

The selection of the reward function *f* is motivated by the following two objectives: (*i*) the vehicle would like to be close to trees so as to hide from “predators,” and (*ii*) it should keep a distance from the trees to avoid potential collisions. We can hypothesize that a moth flying in a forest has some of these objectives as well.

### Refining the moth control policy

Given the function *f*, we maximize the expected average reward in Eq (3) using Algorithm 1. We will discuss the performance of policies under different reward functions in the following Subsection.

We run the algorithm 1000 times and select the 10 policies with the highest average reward estimates *α*. These policies are then simulated to find the best policy, shown in Table 1. The parameters related to obstacle spatial density are much higher in the optimized policy compared to the policy we learned from the moth data. This suggests that integrating detailed information on the location of obstacles into the control policy can largely improve the performance in navigation, at least as it relates to the reward function we defined. Moreover, the history feature is less important for the refined policy. This indicates that the best control for navigation is not necessarily smooth. Indeed, Table 1 compares the reward for the two policies. The performance of the policy learned by the actor-critic algorithm is significantly better, with an increase of the expected average reward by more than 60%.

### Comparison of the two policies in the experimental forest

**Fig 6:**
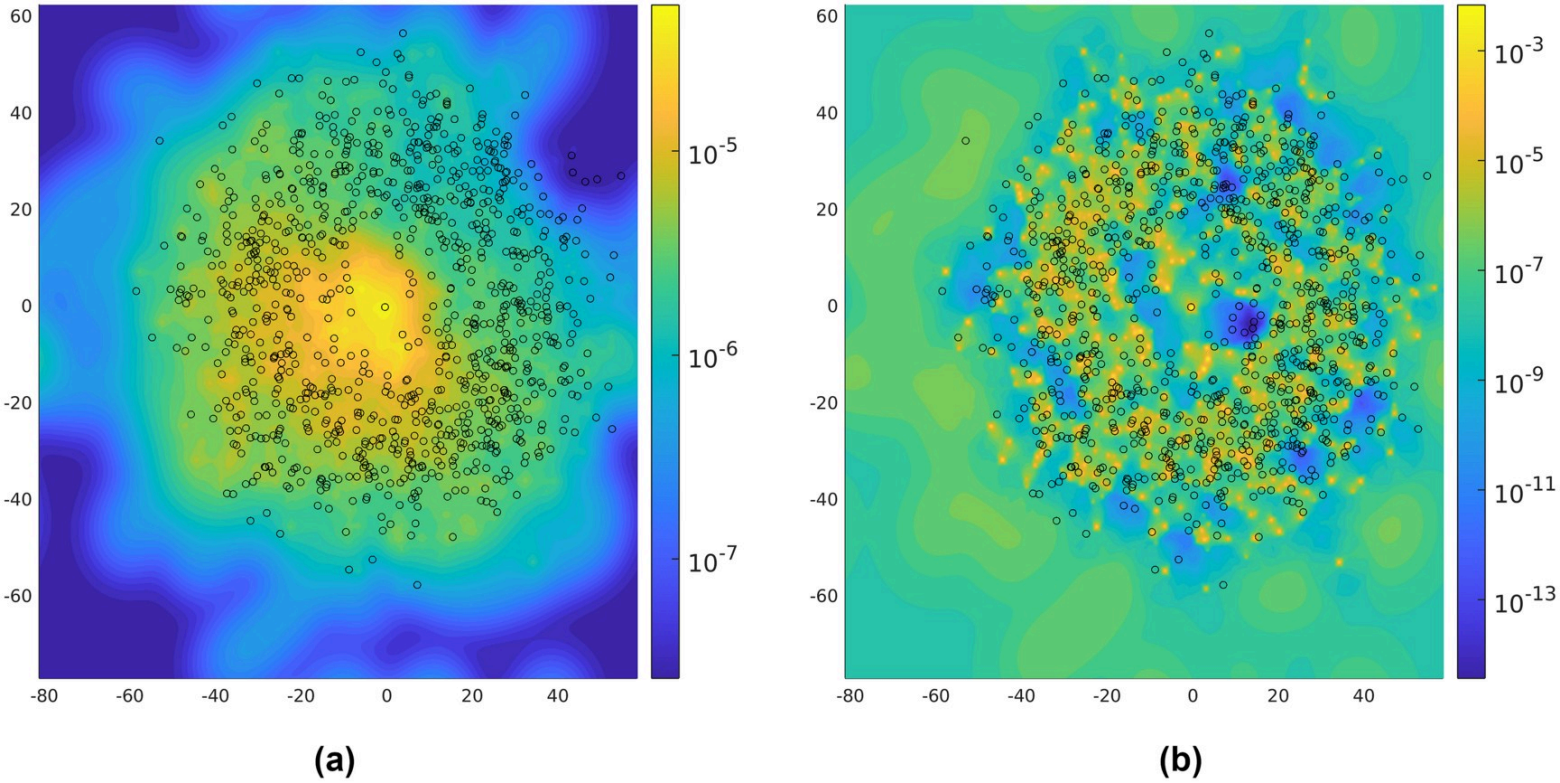
Stationary distribution for the Markov chain {(*x*_*k*_, *y*_*k*_)} under the two policies: (a) the moth policy; (b) the refined policy. In these figures, the black circles indicate trees in the forest.

We next compare the two policies, one regressed by the sparse logistic regression and the other learned by the actor-critic algorithm. We apply both policies in the experimental forest from which the moth data were collected. We aim to calculate the stationary distribution of the Markov chain {**x**_*k*_} under each policy. Since the state-space **x** = {(*x*, *y*, *ω*)} is extremely large, we reduce it using the following approximations. Specifically, we integrate over *ω* and consider the Markov chain whose reduced state is (*x*, *y*) and transition probability from the reduced state (*x*_*k*_, *y*_*k*_) to the reduced state (*x*_*k*+1_, *y*_*k*+1_) under control policy ***θ*** is

$$P\left(x_{k+1},y_{k+1}|x_{k},y_{k}\right)=E_{\omega_{k}∼U}P\left(x_{k+1}=\left(x_{k+1},y_{k+1},\cdot \right)|x_{k}=\left(x_{k},y_{k},\omega_{k}\right),u_{k}∼\mu_{\theta }\right),$$ (11)

where U is a uniform distribution of all possible angles and *μ*_***θ***_ denotes the control policy under parameter ***θ***. In addition, we assume that the control *u*_*k*−1_ is distributed according to the data and the fog level is equal to 6.66 when calculating the features (the same value was used to capture the experimental data). We compute the stationary distribution of the Markov chain {(*x*_*k*_, *y*_*k*_)} according to the above transition probability under the two policies. It is easy to verify that the Markov chains under these policies are irreducible, since the policies are of the Boltzmann type. Therefore, they have unique stationary distributions, depicted in Fig 6.

According to Fig 6, the stationary distribution for the moth policy is smoother than the one for the refined policy. The reward of the agent under each policy is given in Table 1. The performance of the policy learned by the actor-critic algorithm is significantly better than the policy regressed by sparse logistic regression. Comparing the stationary distributions shown in Fig 6, it appears that an agent using the refined policy focuses more intensely in areas with higher reward than an agent using the moth policy. According to Table 1, incorporating obstacle spatial density into the control policy can significantly improve performance in the experimental forest.

### Policy performance in new artificial forests

Next, we construct artificial environments (forests) to investigate how the performance of the moth-based and the refined policy translate to a new environment. We are particularly interested in assessing the fragility (or robustness) of these policies as the environment changes.

**Fig 7:**
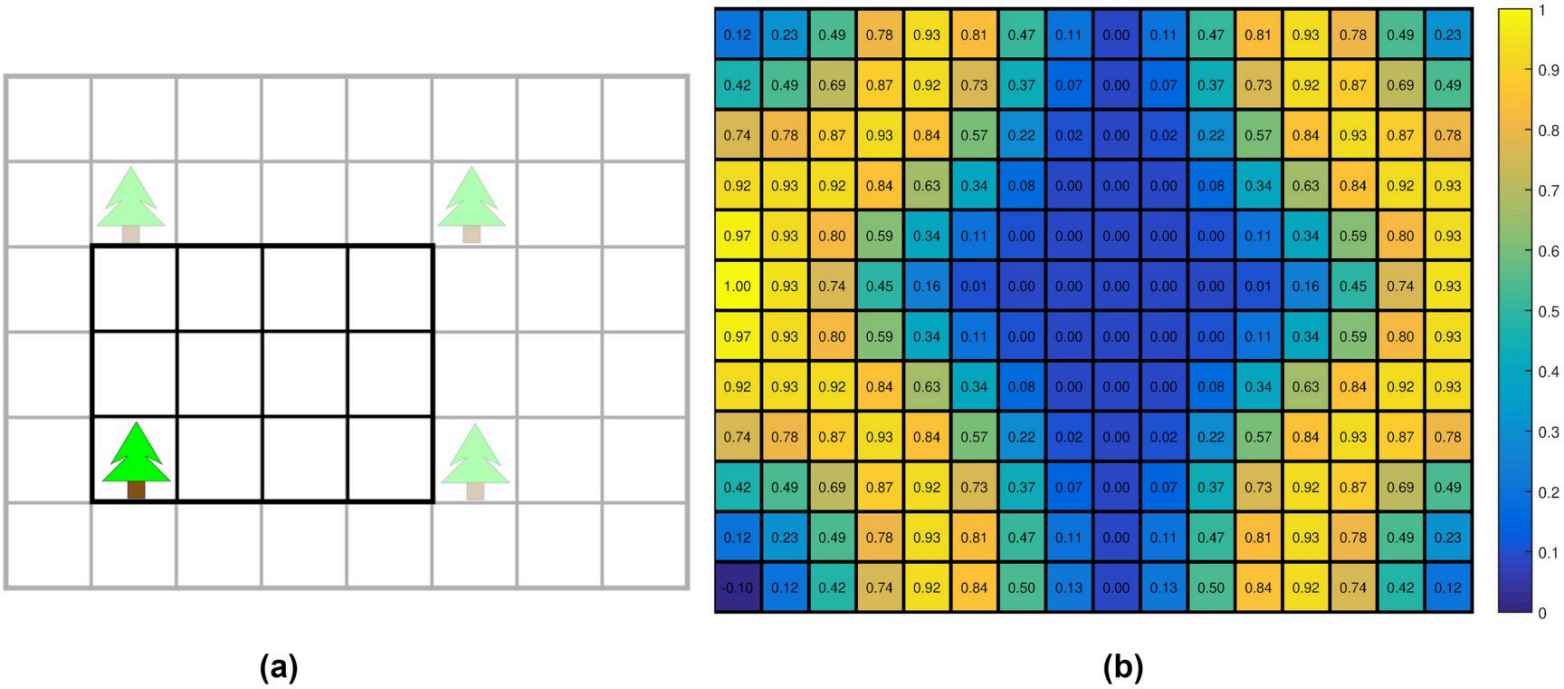
Illustration of artificial forests and their one-step reward function. (a) An example artificial forest. The grid shown in a black solid line is the elementary grid. Repeating this 4 × 3 (*m*^2^) grid forms the artificial forest. (b) An example of the one-step reward distribution in an artificial forest.

As shown in Fig 7(a), a 4 × 3 (*m*^2^) grid with a tree planted at (1, 1) is used as an elementary building block for the artificial forest. This elementary grid is replicated to generate an entire forest. We let the radii of trees in the artificial forests be equal to the average radius of trees in the experimental forest and set the fog level to 6.6. We can control the density of the trees by selecting different sizes of the elementary grid. Moreover, the number of states of the MDP can be reduced by only considering an agent in the elementary grid due to the periodic boundary condition of the MDP. To simulate the agents in such environments, some virtual trees outside the grid should be taken into account when generating the one-step reward functions and calculating obstacle spatial density and optical flow. For example, Fig 7(b) is a one-step reward function for a 16 × 12 gird.

**Fig 8:**
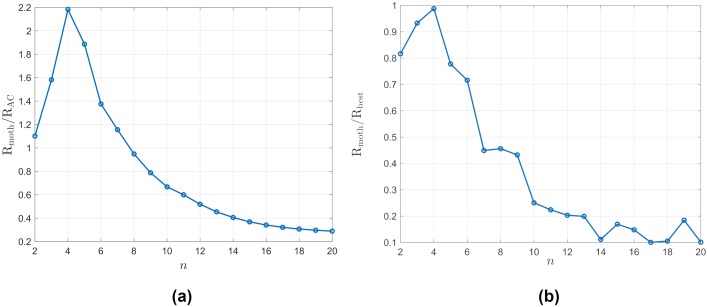
The performance of the moth policy in the artificial forests. (a) Comparing the moth policy and the refined policy in the artificial forests. The *y*-axis plots the ratio of R_moth_/R_AC_. The elementary grids are set to be 4*n* × 3*n*, where *n* = 2, 3, …, 20. (b) Comparing the moth policy and the best policy in the artificial forests. The *y*-axis plots the ratio of R_moth_/R_AC_.

We investigate the performance of the two policies when the density of the trees in artificial forests changes. The elementary grids are set to be 4*n* × 3*n*, where *n* = 2, 3, …, 20. Denote by R_moth_ the expected average reward for agents using the policy learned from the moths by sparse logistic regression, and by R_AC_ the average reward of the actor-critic-based policy. We can think of the latter as a policy with the same structure as the moth policy, trained in the same experimental forest the moths flew, but with parameters optimized to maximize the average reward we defined. We use the ratio R_moth_/R_AC_ to characterize the performance of the moth policy. The ratio is greater than one if the moth policy performs better and less than one if the refined policy is better. The result is shown in Fig 8(a). It can be seen that there exist values of *n* for which the moth policy outperforms the actor-critic-based policy. Recall that the latter has been optimized for the experimental forest the moths flew. The implication is that the moth policy behaves better across a range of artificial forests compared to the actor-critic-based policy.

Next, we compare the performance of the moth policy and the best policy in artificial forests (with an average reward R_best_). The elementary grids are again set to be 4*n* × 3*n*, where *n* = 2, 3, …, 20. The best policy in each forest is calculated by the actor-critic algorithm for each artificial forest (see the discussion in the section on Refining the Moth Control Policy). We compare the expected average rewards of the moth policy and the best policy in each forest in Fig 8(b). As shown in this figure, there exists a certain density of the forests at which the learned moth policy behaves very similar to the best policy. Plausibly, such a density is similar to one encountered by the moths used in the experiment, which would imply that they have implicitly optimized their navigation policy.

### Policy performance as a function of the one-step reward

**Fig 9:**
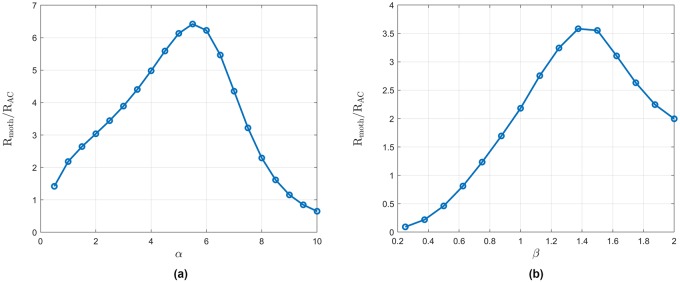
The comparison of the two policies using different *β* (plot (b)) and *α* (plot (a)). The *y*-axis plots the ratio of R_moth_/R_AC_. The elementary grid is set to be 16 × 12 (*m*^2^).

Finally, we change the parameters of the one-step reward function to assess the change in the performance of the two policies (the moth policy and the refined policy by the actor-critic algorithm). The artificial forest is fixed as a 16 × 12 (*m*^2^) grid, where the moth policy performs best. Recall that the one-step reward is generated by the polynomial function *f*_*α*,*β*_. We first fix the parameter *β* = 1 and change *α*. As shown in Fig 9(a), when *α* increases, the performance of the moth policy varies. When *α* becomes too large, the two policies become indistinguishable, since it is hard to find areas in the forest with higher one-step reward. Next we fix the parameter *α* = 1 and change *β*. The result is shown in Fig 9(b). When *β* becomes smaller, the refined policy performs better. This is similar to the situation where the tree density decreases.

## Discussion

In this paper, moth navigation data are utilized to extract a navigation control policy. We model the dynamics of the moth movement and introduce an MDP setting to capture their navigational decision making. We learn a policy consistent with the experimental data using sparse logistic regression. Based on the results in Fig 4 and Table 1, we deduce that the moths do not favor obstacle spatial density in navigation, but heavily rely on optical flow. Indeed, we should not be surprised by these results: optical flow has been demonstrated to be a key factor underlying the control of flight responses in insects [24].

After obtaining the moth control policy, we use it as a starting point for actor-critic algorithms to find the best policy that achieves maximum average reward (in the same experimental forest). The reward function was defined in a way that is consistent with how we may want to guide UAVs flying in similar forest terrains. Such an optimized policy is described in Table 1. We observe that by integrating obstacle spatial density into the control policy, we can improve the performance in navigation.

The moth policy is observed in the experimental forest moths flew and our refined actor critic-based policy is optimized for the same forest. We examined the sensitivity of these policies to changing forest environments. It turns out that the moth policy performs better when the trees in the forest are more dense. Moreover, the moth policy performs best in an artificial forest constructed with an elementary grid 16 × 12 (*m*^2^). One possible explanation is that the moths have optimized their strategy to fit forests similar to what they encounter in their natural habitat.

Admittedly, due to the design of the experiment, the control policy learned from the data may differ from the navigation policy moths could use in a natural forest. From a behavioral ecology perspective, the moths are presumably looking for mates and food; but, under natural conditions, that search behavior would be heavily dependent on olfactory (rather than visual) cues. In addition, moths in nature may not experience foggy conditions. We could plausibly (but without direct evidence) suggest that the navigation behaviors observed might be similar to pseudo-aimless wanderings that a moth might perform when trying to find odor plumes in a natural environment. Nevertheless, no matter what the conditions, we have developed a method to infer a policy consistent with the observed moth data (in our case, obtained in a specific experimental setup) and refine it to obtain a bio-inspired UAV navigation policy. Comparing the moth policy with the best policy, optimized separately for each artificial forest, we observe that the best policy performs better in the specific forest it is optimized for but the original (moth) policy learned from the moth data is quite robust across a number of different forest configurations. Specifically, for an entire range of artificial forests with density in the interval [1/48, 1/432] trees/*m*^2^ (the two endpoints differ by a factor of 9!) the moth policy remains within 30% of the performance of the best policy in each forest. This could be a desirable feature for animals as fast adaptivity to various environments may be critical for survival.

We also examined how the moth policy we learn from the data fares against the optimized policy when we vary the structure of the one-step reward function. Our analysis reveals that the performance of the moth policy is relatively stable when one-step reward parameters *α* and *β* change. This again demonstrates a tradeoff between optimality and robustness.

There may be additional reasons for moths to use the type of policy we estimate. Our analysis shows that the moth navigation policy is better when trees in the forests are more dense. This is consistent with the fact that optical flow variations are considerable larger in denser forests. In addition, when moths navigate in a foggy environment, the lack of visibility leads them to more conservative (hence, further from optimal) decision making.

As shown in this paper, a policy learned from demonstrations is useful for finding an effective navigation policy. In principle, one could obtain a policy from scratch, without using data. However, to that end, one has either to solve a dynamic programming problem, or some approximate variant (e.g., applying the actor-critic method we used in this paper). The former suffers from the well known curse-of-dimensionality and becomes computational intractable for large state-action spaces. The latter approximate approach does not guarantee convergence to an optimal policy but just a local minimum of the policy parameter vector. Moreover, designing an approximation (of the policy function as in Eq (2) or the value function) requires intuition about important features. The key contribution of using demonstration data is that we can identify the most appropriate features the policy approximation should use. The procedure of learning from experts and refining the learned policy we developed in this work is expected to find applications in UAV navigation, especially in navigating relatively dense areas such as forests or urban landscapes featuring many tall buildings and resulting in “urban canyons”.

## Supporting information

S1 Text BFGS Quasi-Newton Method for the logistic regression. (PDF)
