# Supplementary material for: Learning from animals: How to Navigate Complex Terrains
Source: PLoS Comput Biol. 2020 Jan 9;16(1):e1007452. doi: 10.1371/journal.pcbi.1007452 (PMC6952082; doi:10.1371/journal.pcbi.1007452)
Supplement: S1 Text — (PDF) [file pcbi.1007452.s001.pdf]

Learning from Animals: How to Navigate Complex Terrains

Henghui Zhu [1], Hao Liu [2], Armin Ataei [1], Yonatan Munk [3], Thomas Daniel [3], and Ioannis Ch. Paschalidis [*4],

1 Center for Information and Systems Engineering, Boston University, Boston, MA, USA
2 College of Control Science and Engineering, Zhejiang University, Hangzhou, China
3 Department of Biology, University of Washington, Seattle, WA, USA
4 Department of Electrical and Computer Engineering, Division of Systems Engineering, and Department of Biomedical Engineering, Boston University, Boston, MA, USA

* yannisp@bu.edu

S1 Text

BFGS Quasi-Newton Method for the Logistic Regression. This section briefly introduces the BFGS quasi-Newton method for optimization problems and then calculates the gradient of the negative log-likelihood function (cf. Eq. (5) in main paper). For highly readable introductions to quasi-Newton methods, see e.g., [1, 2].

To find the solution of

$$\arg\min_{\boldsymbol{\theta}} \ \text{NLL}(\boldsymbol{\theta}), \tag{S.1}$$

the quasi-Newton method applies the following iteration:

$$\boldsymbol{\theta}^{k+1} = \boldsymbol{\theta}^k - \delta^k \mathbf{D}^k \nabla \text{NLL}(\boldsymbol{\theta}^k),$$

where δ^k is a stepsize and $\mathbf{D}^k$ a scaling matrix updated as follows

$$\begin{aligned}
\mathbf{p}^k &= \boldsymbol{\theta}^{k+1} - \boldsymbol{\theta}^k, \\
\mathbf{q}^k &= \nabla \text{NLL}(\boldsymbol{\theta}^{k+1}) - \nabla \text{NLL}(\boldsymbol{\theta}^k), \\
\tau^k &= (\mathbf{q}^k)' \mathbf{D}^k \mathbf{q}^k, \\
\mathbf{v}^k &= \frac{\mathbf{p}^k}{(\mathbf{p}^k)'\mathbf{q}^k} - \frac{\mathbf{D}^k \mathbf{q}^k}{\tau^k}, \\
\mathbf{D}^{k+1} &= \mathbf{D}^k + \frac{\mathbf{p}^k (\mathbf{p}^k)'}{(\mathbf{p}^k)'\mathbf{q}^k} - \frac{\mathbf{D}^k \mathbf{q}^k (\mathbf{q}^k)' \mathbf{D}^k}{(\mathbf{q}^k)' \mathbf{D}^k \mathbf{q}^k} + \tau^k \mathbf{v}^k (\mathbf{v}^k)'.
\end{aligned}$$

We calculate the gradient of the negative log-likelihood function as follows. Let $h(\mathbf{x}, u) = \exp(\boldsymbol{\theta}' \boldsymbol{\phi}(\mathbf{x}, u))$ for any $u \in \mathbb{U}$ and $H(\mathbf{x}) = \sum_{u \in \mathbb{U}} h(\mathbf{x}, u)$. We have,

$$\begin{aligned}
\nabla_{\boldsymbol{\theta}} h(\mathbf{x}, u) &= \exp(\boldsymbol{\theta}' \boldsymbol{\phi}(\mathbf{x}, u)) \boldsymbol{\phi}(\mathbf{x}, u), \\
\nabla_{\boldsymbol{\theta}} H(\mathbf{x}) &= \sum_{u \in \mathbb{U}} \exp(\boldsymbol{\theta}' \boldsymbol{\phi}(\mathbf{x}, u)) \boldsymbol{\phi}(\mathbf{x}, u).
\end{aligned}$$

As a result,

$$\begin{aligned}\nabla_{\boldsymbol{\theta}}\mu(u_i|\mathbf{x}_i;\boldsymbol{\theta}) &= \nabla_{\boldsymbol{\theta}}\left(\frac{h(\mathbf{x}_i,u_i)}{H(\mathbf{x}_i)}\right) \\ &= \frac{\nabla_{\boldsymbol{\theta}}h(\mathbf{x}_i,u_i)}{H(\mathbf{x}_i)} - \frac{h(\mathbf{x}_i,u_i)\nabla_{\boldsymbol{\theta}}H(\mathbf{x}_i)}{(H(\mathbf{x}_i))^2} \\ &= \mu(u_i|\mathbf{x}_i;\boldsymbol{\theta})\boldsymbol{\phi}(\mathbf{x}_i,u_i) - \mu(u_i|\mathbf{x}_i;\boldsymbol{\theta})\frac{\nabla_{\boldsymbol{\theta}}H(\mathbf{x}_i)}{H(\mathbf{x}_i)} \\ &= \mu(u_i|\mathbf{x}_i;\boldsymbol{\theta})\left[\boldsymbol{\phi}(\mathbf{x}_i,u_i) - \sum_{u\in\mathbb{U}}\mu(u|\mathbf{x}_i;\boldsymbol{\theta})\boldsymbol{\phi}(\mathbf{x}_i,u)\right]. \end{aligned} \tag{S.2}$$

Hence, the gradient of the negative log-likelihood function is

$$\begin{aligned}\nabla_{\boldsymbol{\theta}}\mathrm{NLL}(\boldsymbol{\theta}) &= -\sum_{i=1}^{N}\frac{\nabla_{\boldsymbol{\theta}}\mu(u_i|\mathbf{x}_i;\boldsymbol{\theta})}{\mu(u_i|\mathbf{x}_i;\boldsymbol{\theta})} \\ &= -\sum_{i=1}^{N}\left[\boldsymbol{\phi}(\mathbf{x}_i,u_i) - \sum_{u\in\mathbb{U}}\mu(u|\mathbf{x}_i;\boldsymbol{\theta})\boldsymbol{\phi}(\mathbf{x}_i,u)\right]. \end{aligned} \tag{S.3}$$

In addition, for the sparse logistic regression problem (cf. Eq. (7) in main paper), the objective function $\mathrm{NLL}_{\mathrm{sp}}(\boldsymbol{\theta})$ is not differentiable everywhere. In this case, the subgradient of $\mathrm{NLL}_{\mathrm{sp}}(\boldsymbol{\theta})$ can be used in the BFGS quasi-Newton method. Similar to Eq. (S.3), a subgradient of $\mathrm{NLL}_{\mathrm{sp}}(\boldsymbol{\theta})$ is

$$\tilde{\nabla}_{\boldsymbol{\theta}}\mathrm{NLL}_{\mathrm{sp}}(\boldsymbol{\theta}) = \sum_{i=1}^{N}\sum_{u\in\mathbb{U}}\mu(u|\mathbf{x}_i;\boldsymbol{\theta})\boldsymbol{\phi}(\mathbf{x}_i,u) - \sum_{i=1}^{N}\boldsymbol{\phi}(\mathbf{x}_i,u_i) + \lambda\sum_{j=1}^{n}\mathrm{sgn}(\theta_j).$$

References

1. Bertsekas DP. Nonlinear Programming. 2nd ed. Belmont, MA: Athena Scientific; 1999.

2. Nocedal J, Wright S. Numerical optimization. Springer Science & Business Media; 2006.
